# Socioeconomic Determinants of Ciprofloxacin-Resistant *Shigella* Infections in Bangladeshi Children

**DOI:** 10.20411/pai.v2i1.174

**Published:** 2017-03-25

**Authors:** Randon J. Gruninger, Russell A. Johnson, Sumon K. Das, Eric J. Nelson, Emily S. Spivak, John R. Contreras, A.S.G. Faruque, Daniel T. Leung

**Affiliations:** 1 Division of Infectious Diseases, University of Utah School of Medicine, Salt Lake City, Utah; 2 Westminster College, Masters of Public Health Program, Salt Lake City, Utah; 3 Centre for Nutrition and Food Security, International Center for Diarrhoeal Disease Research, Bangladesh (icddr,b), Dhaka, Bangladesh; 4 Department of Pediatrics, Stanford University School of Medicine, Stanford, California; 5 Emerging Pathogens Institute, Department of Pediatrics, University of Florida, Gainesville, Florida (current); 6 Division of Microbiology and Immunology, University of Utah School of Medicine, Salt Lake City, Utah

**Keywords:** Socioeconomic determinants, *Shigella*, antimicrobial resistance, diarrheal disease

## Abstract

**Background:**

*Shigella* species (spp.) are a leading cause of moderate to severe diarrhea in children worldwide. The recent emergence of quinolone-resistant *Shigella* spp. gives cause for concern, and South Asia has been identified as a reservoir for global spread. The influence of socioeconomic status on antimicrobial resistance in developing countries, such as those in South Asia, remains unknown.

**Methods:**

We used data collected from 2009 to 2014 from a hospital specializing in the treatment of diarrhea in Dhaka, Bangladesh, to determine the relationship between ciprofloxacin-resistant *Shigella* spp. isolates and measures of socioeconomic status in Bangladeshi children less than 5 years of age.

**Results:**

We found 2.7% (230/8,672) of children who presented with diarrhea had *Shigella* spp. isolated from their stool, and 50% (115/230) had resistance to ciprofloxacin. Using multivariable logistic regression analysis, we found that children from families where the father's income was in the highest quintile had significantly higher odds of having ciprofloxacin-resistant *Shigella* spp. compared to children in the lowest quintile (OR = 6.1, CI 1.9-19). Factors protective against the development of resistance included access to improved sanitation (OR = 0.27, CI 0.11-0.7), and improved water sources (OR = 0.48, CI 0.25-0.92). We did not find a relationship between ciprofloxacin resistance and other proxies for socioeconomic status, including the presence of animals in the home, nutritional status, paternal education level, and the number of family members in the home.

**Conclusions:**

Although the associations between wealth and antimicrobial resistance are not fully understood, possible explanations include increased access and use of antibiotics, greater access to healthcare facilities and thus resistant pathogens, or greater consumption of commercially produced foods prepared with antibiotics.

## INTRODUCTION

Antimicrobial resistance (AMR) undermines one of the most important advances in modern medicine and is recognized as one of the greatest threats to global health [1]. Resistance to first-line antibiotics has led to increased morbidity and mortality due to infectious diseases in countries of all income levels. This problem is accentuated in low-income countries where antibiotic use is difficult to control, the population is exposed to a higher frequency of infectious diseases, the quality of antibiotics may be poor, and duration of treatment may be suboptimal [4].

Although there is increased awareness of AMR, limited data exists regarding the impact of socioeconomic status (SES) on AMR patterns. A study conducted in the Amazonian region of Peru found that children from the wealthiest quartile had significantly higher odds of fecal colonization with AMR *Escherichia coli* compared to the least wealthy quartile [5]. However, the study was limited in scope as wealth and education were the only proxies used to assess SES. A recent study described how increased health care costs in low- and middle-income countries have shifted the demand for purchasing antimicrobials to the private, and often unregulated sector [4]. This shift may result in a socioeconomic-dependent barrier that may unequally drive antibiotic resistance. Knowledge of how SES affects antimicrobial resistance patterns is necessary in order to make critically informed interventions targeting this problem in low-income countries.

The impact of SES on clinically relevant enteric infections is not well known. Ciprofloxacin-resistant *Shigella* species (spp.) have emerged over the past decade [6–9], and are an increasingly recognized public health threat in both developed and developing countries, with South Asia being identified as a reservoir for global spread [10]. Several recent studies have implicated *Shigella* spp. as leading etiologic agents of moderate to severe diarrhea in children in South Asia, sub-Saharan Africa, and South America [11, 12]. *Shigella* resistance threatens to worsen an already dire situation for children in developing countries worldwide, and thus there is a need to understand the socioeconomic forces driving resistance. The Dhaka Hospital of the International Centre for Diarrhoeal Disease Research, Bangladesh (icddr,b), is uniquely positioned to study SES and *Shigella* spp. resistance in South Asia; the hospital provides care free of charge to inhabitants of all socioeconomic levels in Dhaka, Bangladesh and also maintains one of the world's largest databases of both bacterial isolates and patient socioeconomic characteristics. Using quinolone-resistant *Shigella* as a model enteric pathogen, the primary objective of our study is to determine socioeconomic predictors of antimicrobial-resistant enteric pathogens in Bangladesh.

## METHODS

### Setting

The icddr,b Dhaka Hospital is an urban treatment center for diarrhea that treats approximately 140,000 patients each year free of charge. Approximately 60% of the patients are children less than 5 years of age [13]. Although patients may have co-morbidities, admission is dependent on presenting with diarrheal disease.

The University of Utah IRB determined that this study does not meet the definitions of Human Subjects Research as data or specimens will not be collected specifically for the currently proposed research project through interaction or intervention with living individuals and no identifiable, individual, or private information is being obtained.

### Surveillance

The Diarrhoeal Disease Surveillance System (DDSS) maintained at the icddr,b has previously been described [14]. In short, the DDSS prospectively collects demographic, clinical, and enteric pathogen data from every 50th patient. As part of this surveillance, a questionnaire is administered to an adult guardian and information is gathered about socio-demographics, medical history, and behaviors. The definitions of key socioeconomic variables used in this study are described in Table 1. Of note, previous antibiotic use was a binary variable with no further detailed history of antibiotic use. A physician then performed a physical examination and collected a stool sample or a rectal swab from each patient who underwent surveillance.

**Table 1.** Definitions of key variables used in the present study

Condition	Definition
**Income Quintile**	Household head (father) monthly income.
**Drinking Water Source**	
**Tap Water**	Primary source of drinking water is tap water
**Non-Tap Water**	Primary source of drinking water is from a tube well, pond, river, or ditch.
**Place of Defecation**	
**Non-Sanitary**	Dug hole, open pit, hanging (defecation platform over a pond, lake, river or other water source), or open defecation (no facility).
**Sanitary/Semi-sanitary**	Flush or pour-flush latrine/toilet with a latrine pit, septic tank, or piped sewer system, or a pit latrine with slab (dry toilet with a raised squatting slab or platform), or a composting toilet (dry toilet designed and maintained to produce inoffensive compost).
**WAZ**	Weight-for-age Z-score
**Normal Weight**	WAZ ≥ -2.00
**Underweight**	WAZ <-2.00

*Shigella* spp. were isolated by conventional culture methods. Antimicrobial testing was performed by the disc diffusion method on Mueller-Hinton agar plates. Susceptibility was determined as recommended by the Clinical Laboratory Standards Institute (CLSI) [15].

### Study design

We conducted a retrospective case-control study to determine risk factors for ciprofloxacin-resistant *Shigella* spp. between children from differing SES. We limited our study to children less than 5 years of age because this population makes up 60% of cases of *Shigella* at our institution, and as a group they are more homogeneous than adults. The DDSS data were extracted between January 2009 and December 2014. The inclusion criteria were children less than 5 years of age and a positive stool culture for *Shigella* spp. Cases were defined as children who had ciprofloxacin-resistant (or reduced-susceptibility) *Shigella* spp. isolates. Controls were defined as children who had ciprofloxacin-susceptible *Shigella* spp. isolates.

### Data analysis

A univariate logistic regression was conducted on each independent variable with the outcome variable, *Shigella* spp. resistance, to identify unconditional associations. Independent variables with a significant *P* value (*P* < 0.10) from the univariate analysis were included in the multivariable logistic regression. For these comparisons, we performed statistical analyses using SAS University Edition (SAS Institute, Cary NC). Statistical significance was defined as a *P* value < 0.05.

## RESULTS

**Figure 1.**
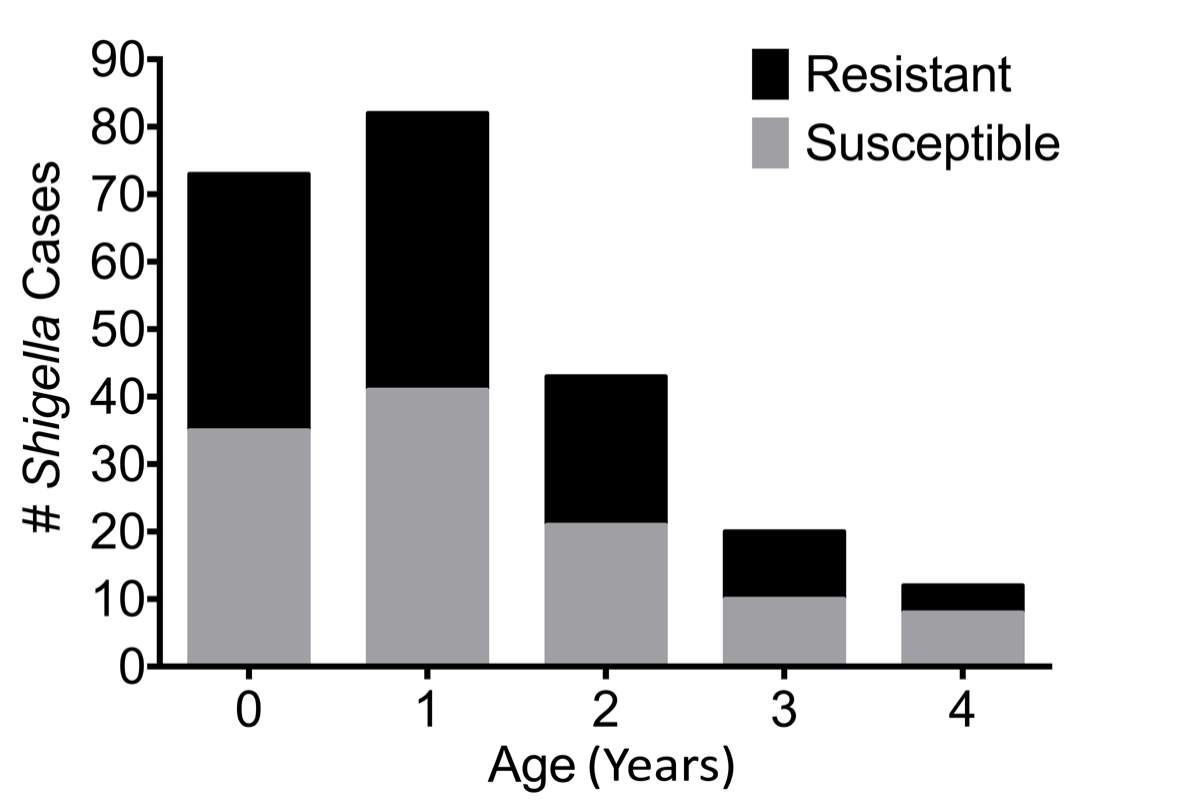
*Shigella* susceptibility by age

Between the years of 2009 to 2014, a total of 16,362 patients were entered into the DDSS. Children less than 5 years of age accounted for 53% (n = 8,672) of patients entered. Among these children, 2.7% (n = 230) had *Shigella* spp. identified in their stools, of which 115 (50%) had either reduced susceptibility or were resistant to ciprofloxacin. From those patients aged 5 or more years, 37% of isolates had either reduced susceptibility or were resistant. The median age of the children was 1 year (IQR = 2) and was not significantly different between cases and controls (Figure 1). For the following analyses, we classified children whose strains were either intermediate or resistant by CLSI guidelines as “resistant”.

**Figure 2.**
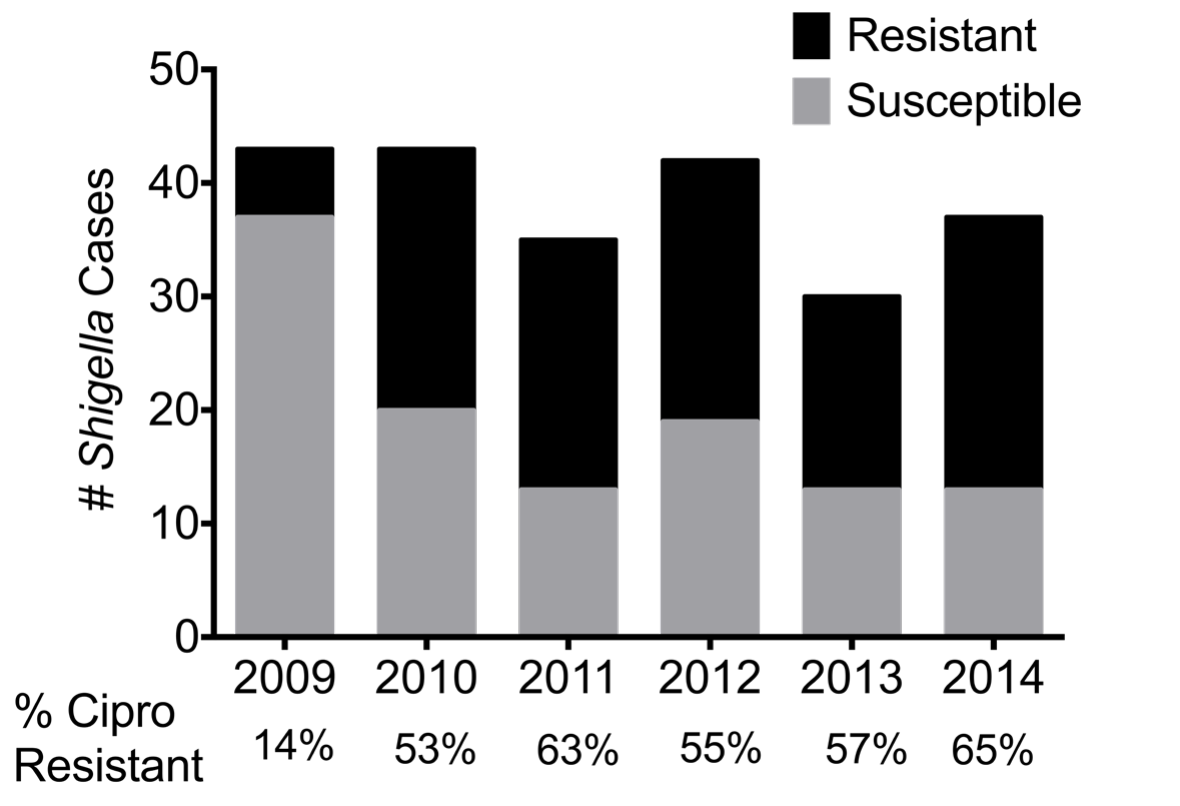
*Shigella* susceptibility by year

We found the 2 most frequent *Shigella* spp. were *Shigella flexneri* (n = 107) and *Shigella sonnei* (n = 76), accounting for 46% and 33%, respectively (Table 2). A higher proportion of *S. sonnei* isolates were ciprofloxacin resistant (63/76; 83%) than S. *flexneri* (52/107, *P* < 0.001; 49%), S. *dysenteriae* (1/10, *P* < 0.001; 10%) or S. *boydii* (0/34, *P* < 0.001). Ciprofloxacin-resistant *Shigella* spp. increased from 14% in 2009 to 53% in 2010. Thereafter, resistance rates have remained relatively stable over time (Figure 2).

**Table 2.** *Shigella* species by susceptibility

Isolate	Cipro S (n = 117)	Cipro R/I (n = 116)
*Shigella flexneri*	55	52
*Shigella sonnei*	13	63
*Shigella* spp.	6	0
*Shigella dys.* II	6	0
*Shigella dys.* 3-12	3	0
*Shigella dys.* 13-15	0	1
*Shigella boydii*	10	0
*Shigella boydii* 7-11	5	0
*Shigella boydii* 12-15	16	0
*Shigella boydii* 16-18	3	0

We conducted a univariate logistic regression to identify unconditional associations (Table 3). We found that 68% (28/41) of children from the highest economic quintile (calculated from the father's income) harbored ciprofloxacin-resistant species compared to 38% (15/40) of children with resistance in the lowest economic quintile (*P* = 0.006). A larger proportion of children who took antibiotics before arriving at the hospital harbored ciprofloxacin-resistant Shigella (*P* = 0.09).

**Table 3.** Univariate Logistic Regression

Variable		Cipro S (%) N = 118	Cipro R/I (%) N = 122	*P*-value
Age, Median (IQR)		1 (2)	1 (2)	0.42
Sex, Female		44 (40)	55 (48)	0.19
Father's Education				
0-5 yrs.		43 (39)	42 (37)	Ref
6-12 yrs.		42 (38)	49 (43)	0.56
>12 yrs.		26 (23)	23 (20)	0.78
Income Quintile	$USD/Year			
Lowest	(0-80)	25 (23)	15 (13)	Ref
Lower Middle	(80-114)	19 (17)	22 (19)	0.15
Middle	(114-189)	33 (29)	31 (27)	0.28
Upper Middle	(189-315)	21 (19)	18 (16)	0.44
**Highest**	(>315)	**13 (12)**	**28 (25)**	**0.006**
Family Members, Median (IQR)		4 (2)	3 (3)	0.24
Antibiotics Before Arrival				
Yes		34 (31)	50 (44)	Ref
**No**		**36 (32)**	**30 (26)**	**0.09**
Unknown		41 (37)	34 (30)	0.08
Drinking Water Source				
**Tap**		**81 (73)**	**65 (57)**	**0.01**
Drinking Water				
Treated		60 (54)	56 (49)	0.46
Place of Defecation				
**Sanitary/Semi-sanitary**		**98 (88)**	**88 (77)**	**0.03**
WAZ				
Normal Weight		68 (61)	83 (73)	Ref
**Underweight**		**43 (39)**	**13 (11)**	**0.07**
Floors, Cemented		92 (83)	83 (73)	0.45
Walls, Brick		84 (76)	77 (68)	0.18
Roofs, Concrete		39 (35)	44 (39)	0.59
Goat in Kitchen, Yes		55/64 (86)	71/84 (85)	0.61
Chicken in Kitchen, Yes		10/11 (91)	22/23 (96)	0.59

To determine the magnitude of association, we included variables with *P* < 0.10 from the univariate analysis in a multivariate logistic regression (the probability modeled is ciprofloxacin = resistance) (Table 4). We found that children from the highest quintile had significantly higher odds of having resistant *Shigella* compared to the children from the lowest quintile (OR 6.1, CI 1.9-19, *P* = 0.002). Children that defecated in a sanitary or semi-sanitary fashion had significantly lower odds of having resistance compared to children that defecated in a hole that had been dug, an open pit, or hanging (OR 0.27, CI 0.11-0.7). Children whose drinking water source was tap water had significantly lower odds of having resistance compared to children whose drinking water source was a tube well, pond, river, or ditch (OR 0.48, CI 0.25-0.92, *P* = 0.03). Exposure to antibiotics prior to hospital arrival was no longer statistically significant after adjusting for covariate interaction and confounders in the multivariate logistic regression model.

**Table 4.** Multivariate logistic regression. Probability modeled is Ciprofloxacin = Resistance

Risk Factor	Odds Ratio	[95% CI]	*P*-value
Age, Median (IQR)	0.83	.64-1.1	0.14
Sex			
Male	Ref	Ref	Ref
**Female**	**2.0**	**1.1-3.6**	**0.03**
Income Quintile			
Lowest	Ref	Ref	Ref
**Lower Middle**	**3.5**	**1.3-9.8**	**0.02**
Middle	2.5	0.93-6.6	0.07
Upper Middle	2.1	0.73-6.2	0.17
**Highest**	**6.1**	**1.9-19**	**0.002**
Antibiotics Before Arrival			
Yes	Ref	Ref	Ref
No	0.99	0.48-2.1	0.51
Unknown	0.65	0.32-1.3	0.22
Drinking Water Source			
Non-Tap Water	Ref	Ref	Ref
**Tap Water**	**0.48**	**0.25-0.92**	**0.03**
Place of Defecation			
Non-sanitary	Ref	Ref	Ref
**Sanitary/Semi-sanitary**	**0.27**	**0.11-0.7**	**0.007**
WAZ			
Normal Weight	Ref	Ref	Ref
Underweight	0.77	0.4-1.5	0.42

## DISCUSSION

Antimicrobial-resistant (AMR) Shigella spp. are an urgent threat to public health, and global spread of ciprofloxacin-resistance *Shigella* spp. is thought to have originated in South Asia [10]. Despite this threat, factors driving *Shigella* spp. resistance have yet to be discerned, and thus interventions to mitigate these trends have not been attainable. However, SES has been postulated as a driver of AMR in developing countries. Indeed, limited studies have shown an association between wealth, increased use of antimicrobials, and AMR both in developing and developed nations [5, 16]. Accordingly, this study aimed to characterize the relationship between ciprofloxacin-resistant *Shigella* spp. and wealth, access to improved sanitation and water sources, education level, and several other proxies of SES among children at an urban hospital specializing in the treatment of diarrhea in Dhaka, Bangladesh. The study site was chosen specifically because patients of broad socioeconomic levels are treated at this hospital and because of its location in South Asia.

We found that young children from higher-income families are significantly more likely to have ciprofloxacin-resistant Shigella spp. This finding is consistent with a Peruvian study that showed a correlation between wealth and antimicrobial resistance in the Amazonian region [5]. Similarly, a study conducted in Malmö, Sweden demonstrated increased consumption of antimicrobials in children from families with a higher per capita income [16]. In our study, the AMR patterns seen could be driven by higher-income families having the resources available to seek care in the private sector, which is an important risk factor for developing resistance [4]. One hypothesis is that the wealthy are more likely to overuse antibiotics and thus develop resistance. This association between antibiotic use prior to arrival and resistance was found on univariate analysis; however, it was no longer significant with adjusted analyses. While antibiotic use prior to arrival could be an indicator of antibiotic overuse, our study did not evaluate the degree of antimicrobial use prior to the acute diarrheal illness. Alternatively, the increased propensity for wealthy patients to harbor resistant organisms could be driven by an altogether different mechanism. A study in Nepal demonstrated that the frequency of antibiotic-resistant microbial carriage in feces was not associated with contact with health care or antibiotics, but rather the community in which one lived, with people from more urban areas harboring more resistance [17]. Wealthy people may live closer to epicenters of resistance because of their employment in major cities. A mechanism for this resistance could be consumption of commercially produced livestock with increased exposure to antimicrobials rather than animals raised on the land free of these exposures [4, 5]. An additional contributor may be city-dwellers' access to unregulated drug vendors, which may facilitate antibiotic self-medication.

Improved sanitation and water sources were independently associated with decreased resistance, after controlling for income and other factors in the multivariable analysis. Despite being proxy measures of income status, these practices likely decrease the overall risk of exposure to resistant bacteria. In our study we did not find an association between ciprofloxacin-resistant *Shigella* spp. isolated in Bangladeshi children and the number of family members present in a child's home, antibiotic exposure prior to arrival, the type of material used to build the home, the presence of animals in the home, or the patients' nutrition scores. These findings argue that other yet-to-be-determined factors related to SES are increasing *Shigella* spp. resistance in South Asia.

Several factors independent of SES are also likely to contribute to *Shigella* spp. resistance in Bangladesh and South Asia. Use of antimicrobials in agricultural practice has been postulated to contribute to AMR both via contact with treated livestock and consumption of animals treated with antibiotics [4]. Our study could be expanded by investigating the relationship between livestock exposure to antibiotics and AMR in household members exposed to those livestock. Plasmid-mediated quinolone resistance has been described recently and is mediated by a pentapeptide protein repeat family that protects DNA gyrase, one of the targets of fluoroquinolones [6]. In addition, plasmid-mediated resistance to trimethoprim (TMP) has rapidly become one of the widest circulating strains of TMP-resistant Chilean *S. sonnei*, underscoring the importance of plasmids in disseminating *Shigella* spp. resistance [18]. Lastly, AMR is thought to be disseminated in wastewater via incomplete treatment of human waste, ultimately bringing humans in contact with feces contaminated by drug-resistant bacteria [3]. Further studies examining the relationship between proximity to wastewater, SES, and AMR are warranted.

This study has several limitations. Notably, the data collected on prior antimicrobial use was collected as a binary variable, thus it was not possible to account for all previous antimicrobial use prior to acute diarrheal illness. Because we used data from a hospital-based surveillance system, our findings are limited to those who sought medical attention. Data collected on income, housing, sanitation, and prior antibiotic use is self-reported, and as such may include errors in self-observation and social desirability bias. Nevertheless, in a large cohort of children with shigellosis, we have shown an association between higher income and infection with ciprofloxacin-resistant *Shigella*. Our findings emphasize the need for prospective studies regarding antimicrobial dispensing in the private sector in low-income countries as well as the antibiotic purchasing habits of higher-income families.

## References

[1] Huttner A, Harbarth S, Carlet J, Cosgrove S, Goossens H, Holmes A, Jarlier V, Voss A, Pittet D Antimicrobial resistance: a global view from the 2013 World Healthcare-Associated Infections Forum. Antimicrob Resist Infect Control. 2013;2:31 PubMed PMID: 24237856. Pubmed Central PMCID: PMC4131211. doi: 10.1186/2047-2994-2-31 24237856 PMC4131211

[2] World Health Organization. Antimicrobial resistance: global report on surveillance.: WHO; 2014 [cited 2016 March 27]. Available from: http://apps.who.int/iris/bitstream/10665/112642/1/9789241564748_eng.pdf?ua=1

[3] Prestinaci F, Pezzotti P, Pantosti A Antimicrobial resistance: a global multifaceted phenomenon. Pathog Glob Health. 2015;109(7):309–18. PubMed PMID: 26343252. Pubmed Central PMCID: PMC4768623. doi: 10.1179/2047773215Y.0000000030 26343252 PMC4768623

[4] Alsan M, Schoemaker L, Eggleston K, Kammili N, Kolli P, Bhattacharya J Out-of-pocket health expenditures and antimicrobial resistance in low-income and middle-income countries: an economic analysis. Lancet Infect Dis. 2015;15(10):1203–10. PubMed PMID: 26164481. Pubmed Central PMCID: PMC4609169. doi: 10.1016/S1473-3099(15)00149-8 26164481 PMC4609169

[5] Kristiansson C, Grape M, Gotuzzo E, Samalvides F, Chauca J, Larsson M, Bartoloni A, Pallecchi L, Kronvall G, Petzold M Socioeconomic factors and antibiotic use in relation to antimicrobial resistance in the Amazonian area of Peru. Scand J Infect Dis. 2009;41(4):303–12. PubMed PMID: 19253090. doi: 10.1080/00365540902783301 19253090

[6] Azmi IJ, Khajanchi BK, Akter F, Hasan TN, Shahnaij M, Akter M, Banik A, Sultana H, Hossain MA, Ahmed MK, Faruque SM, Talukder KA Fluoroquinolone resistance mechanisms of Shigella flexneri isolated in Bangladesh. PLoS One. 2014;9(7):e102533 PubMed PMID: 25028972. Pubmed Central PMCID: PMC4100904. doi: 10.1371/journal.pone.0102533 25028972 PMC4100904

[7] Bowen A, Hurd J, Hoover C, Khachadourian Y, Traphagen E, Harvey E, Libby T, Ehlers S, Ongpin M, Norton JC, Bicknese A, Kimura A, Centers for Disease Control and Prevention. Importation and domestic transmission of Shigella sonnei resistant to ciprofloxacin - United States, May 2014-February 2015. MMWR Morb Mortal Wkly Rep. 2015;64(12):318–20. PubMed PMID: 25837241. 25837241 PMC4584528

[8] Kim JS, Kim JJ, Kim SJ, Jeon SE, Seo KY, Choi JK, Kim NO, Hong S, Chung GT, Yoo CK, Kim YT, Cheun HI, Bae GR, Yeo YH, Ha GJ, Choi MS, Kang SJ, Kim J Outbreak of Ciprofloxacin-Resistant Shigella sonnei Associated with Travel to Vietnam, Republic of Korea. Emerg Infect Dis. 2015;21(7):1247–50. PubMed PMID: 26079171. Pubmed Central PMCID: PMC4480405. doi: 10.3201/eid2107.150363 26079171 PMC4480405

[9] Taneja N Changing epidemiology of shigellosis and emergence of ciprofloxacin-resistant Shigellae in India. J Clin Microbiol. 2007;45(2):678–9. PubMed PMID: 17122011. Pubmed Central PMCID: PMC1829036. doi: 10.1128/JCM.02247-06 17122011 PMC1829036

[10] Chung The H, Rabaa MA, Pham Thanh D, De Lappe N, Cormican M, Valcanis M, Howden BP, Wangchuk S, Bodhidatta L, Mason CJ, Nguyen Thi Nguyen T, Vu Thuy D, Thompson CN, Phu Huong Lan N, Voong Vinh P, Ha Thanh T, Turner P, Sar P, Thwaites G, Thomson NR, Holt KE, Baker S South Asia as a Reservoir for the Global Spread of Ciprofloxacin-Resistant Shigella sonnei: A Cross-Sectional Study. PLoS Med. 2016;13(8):e1002055 PubMed PMID: 27483136. Pubmed Central PMCID: PMC4970813. doi: 10.1371/journal.pmed.1002055 27483136 PMC4970813

[11] Kotloff KL, Nataro JP, Blackwelder WC, Nasrin D, Farag TH, Panchalingam S, Wu Y, Sow SO, Sur D, Breiman RF, Faruque AS, Zaidi AK, Saha D, Alonso PL, Tamboura B, Sanogo D, Onwuchekwa U, Manna B, Ramamurthy T, Kanungo S, Ochieng JB, Omore R, Oundo JO, Hossain A, Das SK, Ahmed S, Qureshi S, Quadri F, Adegbola RA, Antonio M, Hossain MJ, Akinsola A, Mandomando I, Nhampossa T, Acacio S, Biswas K, O'Reilly CE, Mintz ED, Berkeley LY, Muhsen K, Sommerfelt H, Robins-Browne RM, Levine MM Burden and aetiology of diarrhoeal disease in infants and young children in developing countries (the Global Enteric Multicenter Study, GEMS): a prospective, case-control study. Lancet. 2013;382(9888):209–22. PubMed PMID: 23680352. doi: 10.1016/S0140-6736(13)60844-2 23680352

[12] Platts-Mills JA, Babji S, Bodhidatta L, Gratz J, Haque R, Havt A, McCormick BJ, Mc-Grath M, Olortegui MP, Samie A, Shakoor S, Mondal D, Lima IF, Hariraju D, Rayamajhi BB, Qureshi S, Kabir F, Yori PP, Mufamadi B, Amour C, Carreon JD, Richard SA, Lang D, Bessong P, Mduma E, Ahmed T, Lima AA, Mason CJ, Zaidi AK, Bhutta ZA, Kosek M, Guerrant RL, Gottlieb M, Miller M, Kang G, Houpt ER, Investigators M-EN. Pathogen-specific burdens of community diarrhoea in developing countries: a multisite birth cohort study (MAL-ED). Lancet Glob Health. 2015;3(9):e564–75. PubMed PMID: 26202075. doi: 10.1016/S2214-109X(15)00151-5 26202075 PMC7328884

[13] Ferdous F, Das SK, Ahmed S, Farzana FD, Malek MA, Das J, Latham JR, Faruque AS, Chisti MJ Diarrhoea in slum children: observation from a large diarrhoeal disease hospital in Dhaka, Bangladesh. Trop Med Int Health. 2014;19(10):1170–6. PubMed PMID: 25039966. doi: 10.1111/tmi.12357 25039966

[14] Stoll BJ, Glass RI, Huq MI, Khan MU, Holt JE, Banu H Surveillance of patients attending a diarrhoeal disease hospital in Bangladesh. Br Med J (Clin Res Ed). 1982;285(6349):1185–8. PubMed PMID: 6812801. Pubmed Central PMCID: PMC1500105. 10.1136/bmj.285.6349.1185 PMC1500105 6812801

[15] (CLSI) CaLSI. Performance Standards for Antimicrobial Susceptibility Testing; 20th Informational Supplement (June 2010, Updated), CLSI document M100-S20-U, CLSI, Clinical and Laboratory Standard Institute, Fort Wayne, Ind, USA 2010.

[16] Nilsson P, Laurell MH Impact of socioeconomic factors and antibiotic prescribing on penicillin-non-susceptible Streptococcus pneumoniae in the city of Malmo. Scand J Infect Dis. 2005;37(6-7):436–41. PubMed PMID: 16012003. doi: 10.1080/00365540510037795 16012003

[17] Walson JL, Marshall B, Pokhrel BM, Kafle KK, Levy SB Carriage of antibiotic-resistant fecal bacteria in Nepal reflects proximity to Kathmandu. J Infect Dis. 2001;184(9):1163–9. PubMed PMID: 11598839. doi: 10.1086/323647 11598839

[18] Miranda A, Avila B, Diaz P, Rivas L, Bravo K, Astudillo J, Bueno C, Ulloa MT, Hermosilla G, Del Canto F, Salazar JC, Toro CS Emergence of Plasmid-Borne dfrA14 Trimethoprim Resistance Gene in Shigella sonnei. Front Cell Infect Microbiol. 2016;6:77 PubMed PMID: 27489797. Pubmed Central PMCID: PMC4951496. doi: 10.3389/fcimb.2016.00077 27489797 PMC4951496

